# Lung epithelial tip progenitors integrate glucocorticoid- and STAT3-mediated signals to control progeny fate

**DOI:** 10.1242/dev.134023

**Published:** 2016-10-15

**Authors:** Usua Laresgoiti, Marko Z. Nikolić, Chandrika Rao, Jane L. Brady, Rachel V. Richardson, Emma J. Batchen, Karen E. Chapman, Emma L. Rawlins

**Affiliations:** 1 Wellcome Trust/CRUK Gurdon Institute, Wellcome Trust/MRC Stem Cell Institute, Department of Pathology, University of Cambridge, Cambridge CB2 1QN, UK; 2 Centre for Cardiovascular Science, Queen's Medical Research Institute, University of Edinburgh, Edinburgh EH16 4TJ, UK

**Keywords:** Glucocorticoid, STAT3, Mouse, Human, Lung development

## Abstract

Insufficient alveolar gas exchange capacity is a major contributor to lung disease. During lung development, a population of distal epithelial progenitors first produce bronchiolar-fated and subsequently alveolar-fated progeny. The mechanisms controlling this bronchiolar-to-alveolar developmental transition remain largely unknown. We developed a novel grafting assay to test if lung epithelial progenitors are intrinsically programmed or if alveolar cell identity is determined by environmental factors. These experiments revealed that embryonic lung epithelial identity is extrinsically determined. We show that both glucocorticoid and STAT3 signalling can control the timing of alveolar initiation, but that neither pathway is absolutely required for alveolar fate specification; rather, glucocorticoid receptor and STAT3 work in parallel to promote alveolar differentiation. Thus, developmental acquisition of lung alveolar fate is a robust process controlled by at least two independent extrinsic signalling inputs. Further elucidation of these pathways might provide therapeutic opportunities for restoring alveolar capacity.

## Abstract (precis)

**Highlighted Article:** Glucocorticoid receptor and STAT3 signalling act in parallel, but cross-regulated, pathways to control epithelial differentiation during lung alveolar development in mice.

## INTRODUCTION

The gas exchange capacity of the lung is determined by its functional alveolar surface area. During mouse lung development the early phase [pseudoglandular, around embryonic day (E)12.5-15.5] of branching morphogenesis has been mapped in great detail and produces the bronchiolar (conducting airway) tree (Short et al., 2013). During later morphogenesis (canalicular stage, ∼E16.5-17.5), although the pattern is less well-defined, branching continues to produce the framework for future alveolar development (Alanis et al., 2014). The final size of the gas exchange surface is therefore likely to be strongly influenced by the extent of morphogenesis in the canalicular phase of lung development. Defining the mechanisms that control the developmental transition between bronchiolar and alveolar morphogenesis might ultimately permit manipulation of the size of the alveolar surface for therapeutic purposes.

Definitive lineage-tracing experiments have shown that during lung development the distal tip epithelial cells comprise a multipotent progenitor population (Alanis et al., 2014; Desai et al., 2014; Rawlins et al., 2009a). Tip progenitors are defined by a specific molecular signature, including high levels of SOX9 and ID2. During the pseudoglandular stage their descendants exit the distal tip as SOX2^+^ bronchiolar progenitors and from ∼E16.5, during the canalicular stage, their descendants leave the distal progenitor pool as SOX2^−^ alveolar progenitors. Distal progenitors persist at the edge of the lungs until late E17.5 or early E18.5, after which tip structures can no longer be detected. Recent molecular experiments have shown that from ∼E16.5, distal progenitors express low levels of markers of both type I and II alveolar epithelial cells (AT1 and AT2) (Desai et al., 2014; Treutlein et al., 2014). Maturation of AT1 and AT2 cells probably occurs as SOX2^−^ alveolar progenitors downregulate markers of one lineage whilst upregulating those of the other.

It has been known for many years that glucocorticoid signalling can promote maturation of alveolar cells into functional AT1 and AT2 cells. *Glucocorticoid receptor* (*Nr3c1*, here *GR*) null lungs produce fewer AT1 and AT2 cells and synthetic glucocorticoids are routinely used to promote alveolar maturation in infants at risk of premature birth (Cole et al., 2004). A recent study showed that precocious administration of glucocorticoid during mouse lung development promotes the transition from bronchiolar to alveolar fate in the distal progenitors. Moreover, alveolar initiation was delayed in *GR^−/−^* mutant lungs, resulting in an extra round of bronchiolar branching (Alanis et al., 2014). Hence GR-mediated signalling controls the timing of alveolar initiation. However, GR signalling is not absolutely necessary for distal progenitor alveolar fate, or alveolar differentiation, and additional mechanisms must also regulate these processes.

To establish whether an intrinsic mechanism or external factors trigger the bronchiolar-to-alveolar developmental transition during normal development, we developed a heterochronic grafting assay. These experiments showed that non-cell autonomous signalling plays a major role in determining progeny fate of SOX9^+^ distal tip cells. We investigated the underlying molecular mechanisms and present evidence that STAT3 and GR act in parallel during lung alveolar initiation and are individually sufficient to promote alveolar differentiation.

## RESULTS

### Expression of alveolar fate markers during mouse lung embryonic development

**Fig. 1.**
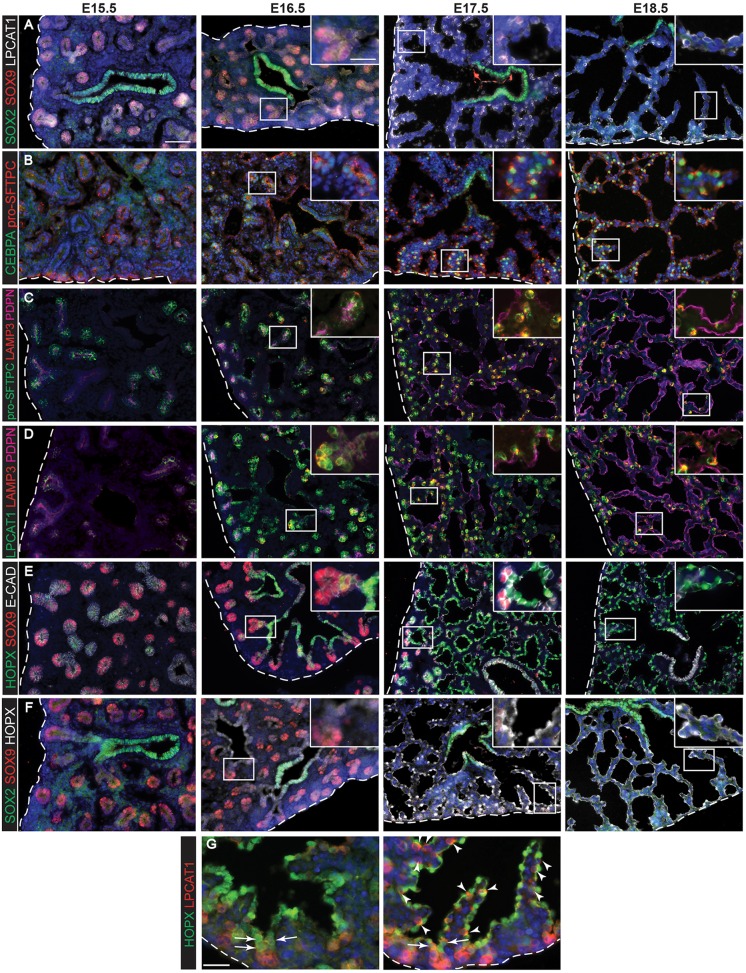
**Evolution of alveolar epithelial gene expression patterns in the developing mouse lung.** Sections of E15.5, 16.5, 17.5 and 18.5 wild-type mouse lungs stained for markers of differentiation. (A) Green, SOX2 (differentiating bronchioles); red, SOX9 (tips); white, LPCAT1 (tip cells from E16.5, then AT2 cells). (B) Green, CEBPA (sub-set of tip cells from E16.5, then AT2 cells); red, pro-SFTPC (embryonic epithelium, stronger from E16.5, later specific to AT2 cells). (C) Green, pro-SFTPC (stronger from E16.5, later specific to AT2 cells); red, LAMP3 (rare tip cells; AT2 cells); magenta, PDPN (tip cells from E16.5, then AT1 cells). (D) Green, LPCAT1 (tip cells from E16.5, then AT2 cells); red, LAMP3 (rare tip cells; AT2 cells); magenta, PDPN (tip cells from E16.5, then AT1 cells). (E) Green, HOPX (stalk cells from E16.5, AT1 cells); red, SOX9 (tip cells); white, E-CAD (epithelial cells). (F) Green, SOX2 (differentiating bronchioles); red, SOX9 (tips); white, HOPX (stalk cells from E16.5, AT1 cells). (G) Green, HOPX (stalk cells from E16.5, AT1 cells); red, LPCAT1 (tip cells from E16.5, then AT2 cells). Arrows, LPCAT1^+^ HOPX^+^ cells; arrowheads, LPCAT1^+^ HOPX^−^ cells. Blue, DAPI (nuclei). Dashed line, edge of lung. Scale bars: 50 μm in A-F, 20 μm in G and insets.

It was recently reported that alveolar gene expression begins in distal tip epithelial progenitors before overt morphological signs of alveolar differentiation (Desai et al., 2014; Jain et al., 2015; Treutlein et al., 2014). We performed an expression time-course of AT1 and AT2 cell markers from E15.5 to E18.5 in wild-type lungs, providing a reference for assessing the extent of alveolar specification and/or differentiation under experimental conditions. SOX2 and SOX9 are well-established markers of the differentiating bronchioles and tip progenitors (Fig. 1A). We observed very low, variable, levels of lysophosphatidylcholine acyltransferase 1 (LPCAT1) in E15.5 lung sections (Fig. 1A). It is then robustly detected in tip progenitors from E16.5 and upregulated further in differentiating AT2 cells, consistent with previous reports (Chen et al., 2006; Nakanishi et al., 2006). This makes LPCAT1 expression a useful marker of alveolar fate in distal tip progenitors.

Pro-surfactant protein C (pro-SFTPC, also known as pro-SP-C) is expressed throughout the lung epithelium from the pseudoglandular stage (Wuenschell et al., 1996). We observed that it is also upregulated in the distal epithelial progenitors at E16.5 and subsequently in differentiating AT2 cells (Fig. 1B). The AT2 cell-specific transcription factor CEBPA (also known as C/EBPα) is first detected in the nucleus of a subset of distal epithelial progenitors from E16.5 and then upregulated in differentiating AT2 cells (Fig. 1B) as previously reported (Martis et al., 2006). Earlier, weaker, expression at E15.5 is not nuclear, making nuclear CEBPA a marker of alveolar fate in the distal progenitors. We also observed nuclear CEBPA staining in the bronchioles from E17.5 (Fig. 1B). A recent report has suggested that CEBPA functions redundantly with CEBPB to promote airway differentiation (Roos et al., 2012). We hypothesize that the airway CEBPA staining we observe reflects a second site of expression and therefore do not use this protein as a specific marker of developing alveolar fate.

Similar to pro-SFTPC, the type 1 cell marker podoplanin (PDPN, also known as T1α in mouse) is weakly expressed in the distal progenitors from E15.5 and upregulated in differentiating AT1 cells (Fig. 1C,D). By contrast, lysosomal associated membrane protein 3 (LAMP3) is expressed strongly in differentiated AT2 cells, but could not be detected robustly in distal progenitors. Rather, at E16.5 LAMP3 is expressed at low levels in cells adjacent to the distal progenitor domain, with levels increasing in these cells at E17.5 (Fig. 1C,D). This makes LAMP3 useful as a marker of early AT2 differentiation, rather than alveolar fate within the distal progenitor population.

The AT1-specific transcription factor, HOP homeobox (HOPX) could not be detected in the distal progenitors. However, it is robustly detected from E16.5 in cells that had exited the distal progenitor domain, but never in the SOX2^+^ differentiating bronchiolar cells (Fig. 1E,F). We noted that cells that have exited the distal progenitor domain by E17.5 reproducibly express either LPCAT1 or HOPX, but not both, suggesting that they are already starting to differentiate along AT1 or AT2 lineages (Fig. 1G). By contrast, cells adjacent to the distal tip at both E16.5 and E17.5 co-express LPCAT1 and HOPX. Relative quantitation of the expression of LPCAT1, LAMP3, HOPX and PDPN (Fig. S1) is in agreement with our descriptions based on visual inspection of the images.

Therefore, and consistent with recent reports, we have found that distal tip progenitors begin to express some protein markers of alveolar fate at ∼E16.5 (CEBPA, LPCAT1, PDPN). By contrast, other protein markers (LAMP3, HOPX) cannot be robustly detected until cells have exited the distal progenitor domain. This timing of alveolar marker expression agrees with the available lineage-tracing data that shows that the distal progenitor cells only produce alveolar-fated (and not bronchiolar-fated) descendants from ∼E16.5 onwards (Alanis et al., 2014; Desai et al., 2014; Rawlins et al., 2009a). We hypothesized that the distal progenitors respond to an extrinsic signalling cue to initiate the alveolar programme of development. Alternatively, the progenitors could be intrinsically, cell autonomously, programmed to produce alveolar progeny from ∼E16.5, similar to the temporal production of specific cell identities from neural stem and/or progenitors in the developing vertebrate nervous system (Livesey and Cepko, 2001).

### Heterochronically grafted lung epithelial distal progenitors can respond to signals from their local environment and alter descendant cell fate

**Fig. 2.**
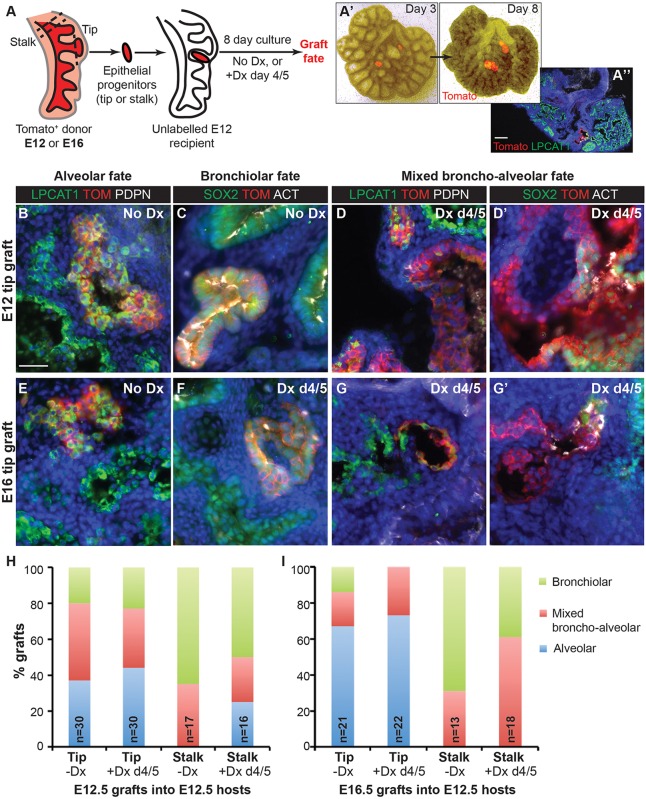
**Extrinsic factors are the major determinant of progenitor cell identity in the developing mouse lung.** (A) Experimental outline. Epithelial progenitors (tip or stalk) were microdissected from donor E12.5 or E16.5 Tomato^+^ (*Rosa26R^mT−mG/+^*) lungs and grafted into the mesenchyme of unlabelled E12.5 hosts. Hosts and grafts were cultured for 8 days without Dx, or with addition of 50 nM Dx at culture day 4 or 5, followed by serial sectioning and staining to determine graft fate. (A′,A″) Grafts integrate into the host lung, grow and form a lumen. (B-G) Sections of grafted lungs with alternate slides stained for: green, LPCAT1 (alveolar fate); red, Tomato (graft); white, PDPN (basal and AT1 cells), or: green, SOX2 (bronchiolar fate); red, RFP (Tomato^+^ graft); white, acetylated tubulin (ACT; cilia) to determine graft fate. Examples of tip grafts with alveolar, bronchiolar and mixed broncho-alveolar fate are shown. Note D/D′ and G/G′ are different sections of the same graft. (H,I) Quantitation of graft fate as a percentage of numbers of grafts analysed. Each type of graft was analysed in at least three independent experiments. Scale bars: 25 μm in A″, 100 μm in B-G′.

To distinguish between our alternative hypotheses for extrinsic versus intrinsic control of distal progenitor fate we asked two related questions. Firstly, is the alveolar fate of the E16.5 SOX9^+^ distal progenitors fixed, or can they respond to local environmental cues, probably from the mesenchyme, and reactivate a bronchiolar pathway? Secondly, can bronchiolar-fated already-differentiating SOX2^+^ stalk cells respond to environmental cues from the mesenchyme and produce alveolar-fated descendants? We microdissected pure populations of tip and stalk cells (Fig. S2), then established a grafting assay to place E12.5 or E16.5 tip or stalk ubiquitous-Tomato^+^ epithelium into the mesenchyme of unlabelled E12.5 host lungs. Hosts were cultured on a membrane to test the response of the grafted cells to their new environment (Fig. 2A). The grafts integrated into the host lungs, were surrounded by host mesenchyme, increased in size over time, and frequently formed a lumen [Fig. 2A′,A″; 220/232 (95%) of differentiated grafts had a lumen]. Although the stalk samples we dissected always contained some adjacent mesenchyme (Fig. S2), we were unable to detect grafted mesenchymal cells at the end of the culture period, suggesting that they did not survive. Grafts were identified based on Tomato expression and scored as bronchiolar-, mixed- (broncho-alveolar), or alveolar-fated by immunostaining alternate slides of serially sectioned host lung and graft for SOX2 and acetylated-tubulin (ACT) (bronchiolar markers), or LPCAT1 and PDPN (alveolar markers). Grafts were scored as mixed if they contained distinct bronchiolar and alveolar regions each greater than 10 cells in size (Fig. 2B-G). Overall differentiation efficiency was 98% (232/237 recovered grafts had differentiated).

As a positive (isochronic) control we grafted E12.5 tips into E12.5 hosts. Addition of a synthetic glucocorticoid, such as dexamethasone (Dx), is necessary to produce differentiated alveolar cells from E12.5 lungs grown in culture. However, Dx has also been shown to promote tip progenitor alveolar fate (Alanis et al., 2014). We therefore cultured host lungs without Dx, or in the presence of Dx from culture day 4 or 5, to allow production of more mature alveolar cells but to minimise precocious alveolar fate specification (Fig. 2A). Previous lineage-labelling experiments showed that ∼80% of individual distal tip cells labelled *in vivo* at E12.5 generate mixed clones of both bronchiolar and alveolar descendants (Rawlins et al., 2009a). In our isochronic control experiments (E12.5 tip grafted into E12.5 host), we obtained ∼20% bronchiolar-, 40% mixed- and 40% alveolar-fated grafts (Fig. 2B-D,H), consistent with the position of the graft influencing cell fate. By contrast, E12.5 stalks grafted into E12.5 hosts were more likely to be bronchiolar-fated, although mixed- and alveolar-fated grafts were also observed (Fig. 2H; Fig. S3) (*P*=0.0075). Moreover, these mixed- and alveolar-fated stalk grafts expressed the AT2-specific marker LAMP3 (Fig. S3H-J). This surprising result suggests that although stalks have initiated a bronchiolar developmental programme, they retain some plasticity to respond to extrinsic cues from the local environment and alter fate. Overall, the results obtained with the two growth conditions (no Dx, or Dx from culture day 4/5) were very similar (Fig. 2H).

We next performed heterochronic grafting experiments in which E16.5 tip or stalk cells were grafted into E12.5 lungs grown without Dx, or with a short period of Dx-exposure (Fig. 2A). Consistent with their developmental age, the E16.5 tip grafts were more likely to produce only alveolar-fated descendants than their E12.5 counterparts (Fig. 2E-G,I; *P*=0.0438). Nevertheless, bronchiolar-fated and mixed broncho-alveolar-fated grafts were also observed, showing that the E16.5 tip progenitors, which normally only produce alveolar descendants, can respond to local environmental cues and change their behaviour appropriately. The E16.5 stalk grafts behave very similarly to the E12.5 stalk grafts in this assay, although with somewhat less plasticity in that they never produce only alveolar-fated descendants (Fig. 2I; Fig. S3).

We conclude that both distal progenitor cells and differentiating stalk cells can respond to their local environment and produce appropriate descendants. This supports the hypothesis that extrinsic signalling, probably from the local mesenchyme, is a major determinant of lung epithelial progenitor cell fate. However, older distal tip and stalk cells become more refractory to external cues with time, presumably corresponding to increased levels of differentiation, suggesting that they have also undergone intrinsic, possibly epigenetic, changes that cell autonomously reduce their capacity to respond to an external signal.

### Glucocorticoid signalling is sufficient, but not necessary, for tip alveolar fate

**Fig. 3.**
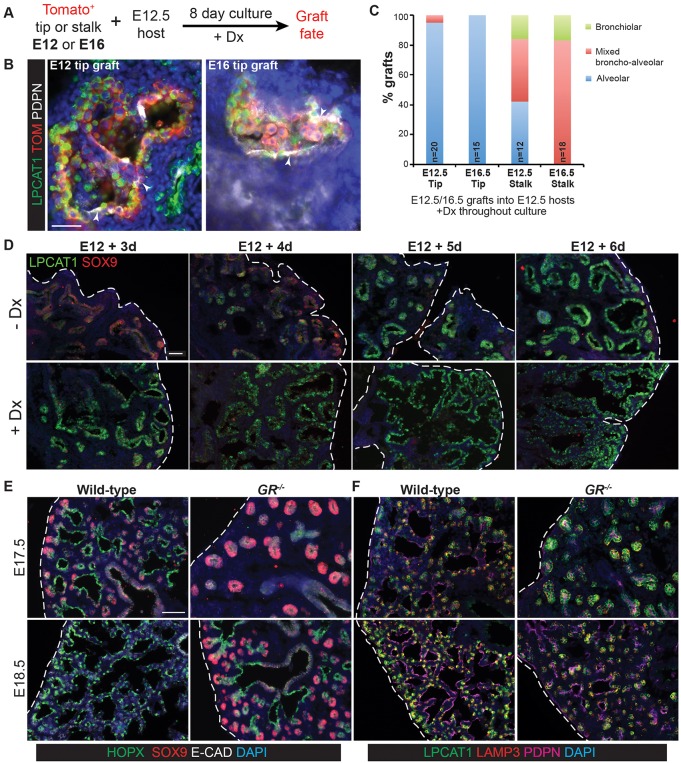
**Glucocorticoid signalling is sufficient, but not essential, to specify alveolar fate.** (A) Experimental design: Tomato^+^ E12.5 or 16.5 tip or stalk was grafted into E12.5 host lung and grown with 50 nM Dx throughout culture. (B) Examples of alveolar-fated tip grafts stained for: green, LPCAT1 (alveolar fate); red, Tomato (graft); white, PDPN (basal and AT1 cells). Arrowheads, PDPN^+^ AT1 cells. (C) Split bar graph showing results from B. Each type of graft was analysed in at least three independent experiments. (D) E12.5 wild-type lungs were grown with or without Dx for up to 6 days; two independent experimental replicates. Note precocious expression of alveolar markers in the presence of Dx. Lungs cultured without Dx do express LPCAT1 from experimental day 5. Green, LPCAT1 (late tip progenitors and type 2 cells); red, SOX9 (tip progenitors). (E,F) Sections of *GR^−/−^* and *GR^+/+^* sibling lungs at E17.5 and E18.5 stained for: green, HOPX (AT1 cells); red, SOX9 (tip progenitors); white, E-CAD (epithelium) (E), and: green, LPCAT1 (late tip progenitors and AT2 cells); red, LAMP3 (AT2 cells); magenta, PDPN (late tip progenitors and AT1 cells) (F). A total of five *GR^−/−^* and 5 *GR^+/+^* sibling lungs from three independent litters were observed at both E17.5 and E18.5. Blue, DAPI. Dashed line, edge of lung. Scale bars: 100 μm in B; 50 μm in D-F.

What are the extrinsic signals that promote alveolar fate in the tip progenitors? A recent report showed that GR signalling, probably induced by circulating glucocorticoids, was sufficient to promote precocious lung alveolar fate both *in vitro* and *in vivo* (Alanis et al., 2014). We therefore tested if GR signalling is also sufficient to promote alveolar fate in our grafting experiments. Host lungs were exposed to Dx throughout the culture period and the fate of the grafts determined (Fig. 3A-C). In these conditions almost 100% of distal tip grafts produced alveolar-fated descendants. Moreover, attenuated PDPN^+^ cells with the appearance of AT1 cells differentiated in these grafts (Fig. 3B), consistent with sustained glucocorticoid signalling being necessary for AT1 differentiation. By contrast, grafted stalk cells were less likely to respond to the signal by producing alveolar descendants (Fig. 3C; *P*=0.0016). However, in the presence of Dx, grafted stalks were more likely to produce alveolar-fated or mixed broncho-alveolar-fated descendants than grafted stalks without Dx, or when Dx was added late in the culture period (compare Fig. 3C with Fig. 2H,I; *P*=0.0067). These experiments confirm the ability of GR signalling to promote alveolar fate. In addition, they show that the undifferentiated distal tip cells are completely plastic in their ability to respond to this signal.

Is GR signalling necessary for tip progenitors to initiate alveolar gene expression? We observed that wild-type E12.5 lungs grown *in vitro* with Dx initiated widespread LPCAT1 expression by culture day 3. By contrast, in the absence of Dx, LPCAT1 was only robustly detected at culture day 5 (Fig. 3D). Thus, addition of Dx promotes and/or accelerates alveolar gene expression, but is not an absolute requirement for alveolar fate initiation to occur *in vitro*. However, it is possible that embryonic lungs can also endogenously synthesise glucocorticoids *de novo* (Boucher et al., 2014). We therefore examined the timing of expression of alveolar fate markers in developing lungs from *GR^−/−^* embryos (Fig. 3E). We observed that, as previously published, the *GR^−/−^* lungs were developmentally delayed. Nevertheless, they did express markers of both tip alveolar fate (LPCAT1, PDPN) and alveolar differentiation (HOPX, LAMP3), albeit delayed, relative to wild-type. This shows that GR signalling affects the timing of alveolar fate acquisition, but is not essential for either tip progenitor alveolar fate induction, or initial alveolar differentiation. We therefore hypothesized that other extrinsic signals acting prior to, or in parallel with, glucocorticoids also promote alveolar fate. Moreover, based on our *in vitro* grafting results we hypothesized that these signals would be expressed within the lung itself.

### Overexpression of *Stat* genes promotes alveolar fate and/or differentiation in cultured lungs

**Fig. 4.**
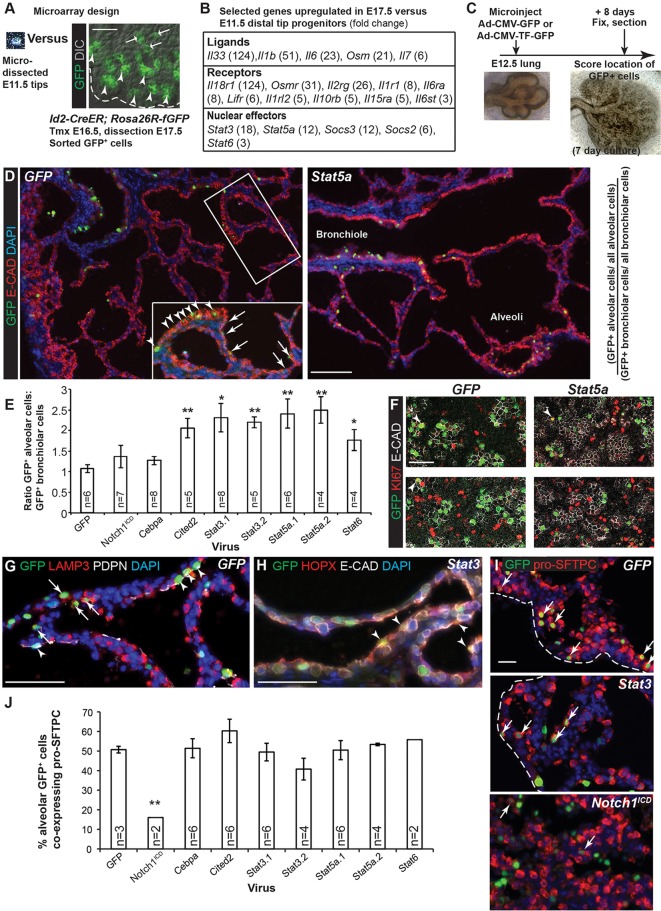
**Overexpression of *Stat* genes promotes alveolar fate in distal tip progenitor cells.** (A) RNA was extracted from microdissected E11.5 distal tips and from flow-sorted GFP^+^ E17.5 tip progenitors (arrowheads), and their immediate alveolar-fated progeny (arrows), from *Id2-CreER; Rosa26R-fGFP* embryos lineage-labelled by tamoxifen (Tmx) injection at E16.5. (B) Selected genes upregulated in E17.5 versus E11.5 samples. Fold-change is shown in brackets. (C) Experimental design for adenoviral-mediated overexpression of TFs in E12.5 lung epithelium. (D) Representative images of *GFP* control and *Stat5a* adenoviral-infected lungs. Green, GFP (transduced cells); red, E-CAD (epithelium). All GFP^+^ and all E-CAD^+^ cells were counted manually. Bronchiolar (arrowheads) versus alveolar (arrows) fate was based on the location, morphology (columnar or squamous) and intensity of the E-CAD staining. (E) Quantification of the ratio of GFP^+^ alveolar:bronchiolar cells normalised to the total numbers of alveolar and bronchiolar epithelial cells scored. (F) Sections of adenovirus-transduced lungs. Green, GFP (transduced cells); red, KI67 (proliferating cells); white, E-CAD (epithelium). Arrowheads, proliferating GFP^+^ epithelial cells. (G) Green, GFP (transduced cells); red, LAMP3 (AT2); white, PDPN (AT1). Arrows, GFP^+^ AT2 cells; arrowheads, GFP^+^ AT1 cells. (H) Green, GFP (transduced cells); red, HOPX (AT1); white, E-CAD (epithelium). Arrowheads, GFP^+^ AT1 cells. (I) Green, GFP (transduced cells); red, pro-SFTPC (AT2 cells). Arrows, co-expressing cells scored as GFP^+^ pro-SFTPC^+^. (J) Quantification of the percentage of alveolar GFP^+^ cells that co-express pro-SFTPC. Blue, DAPI (nuclei). Dashed line, edge of lung. Scale bars: 100 μm in A,D; 50 μm in E,G-I. Error bars represent s.e.m. *P<0.05; **P<0.01 by two-tailed *t*-tests with unequal variance. Full experimental details are presented in the raw data of Table S1.

To search for candidate signalling pathways that control alveolar fate, we isolated E11.5 and E17.5 distal tip cells, with a small number of their immediate progeny, and compared their transcriptomes using gene expression microarrays (Fig. 4A). We performed Gene Ontology (GO) analysis of the genes enriched in E17.5 samples and found that the most prominent GO classes were: Immune System Process; Immune Response; Response to External Stimulus; Response to Wounding. Within these classes it was particularly noticeable that components of cytokine signalling were enriched in the older samples (Fig. 4B). Cytokine signalling is reportedly important for lung maturation (Ikegami et al., 2008; Matsuzaki et al., 2008; Moreno-Barriuso et al., 2006). We therefore tested various cell-autonomous transcription factors (TFs) for their ability to promote alveolar fate *in vitro* with a focus on STAT proteins, which are important mediators of cytokine signalling. Adenoviruses carrying GFP, or a TF+GFP, were microinjected into the lumen of wild-type E12.5 lungs. This resulted in labelling of both tip and stalk cells within 72 h. Lungs were then cultured for 8 days in the presence of Dx, fixed and sectioned, and the location of GFP^+^ cells scored as alveolar or bronchiolar (Fig. 4C).

GFP^+^ cells transduced by control adenovirus were equally likely to be located in the alveoli or bronchioles (normalised GFP^+^ alveolar:bronchiolar ratio of 1; Fig. 4D,E). This was unchanged by notch1 intracellular domain (NOTCH1^ICD^), consistent with previous *in vivo* results (Guseh et al., 2009). Similarly, CEBPA, which is expressed in E16.5 distal progenitors and also in bronchiolar cells from E17.5 (Fig. 1B), was unable to affect the location of GFP^+^ cells. By contrast, we found that over-expression of STAT3, 5A or 6, or CITED2, a TF required for AT2 differentiation (Xu et al., 2008a), was each sufficient to increase the GFP^+^ alveolar:bronchiolar ratio to ∼2 (Fig. 4E). Dividing GFP^+^ cells were detected only rarely in our cultures (Fig. 4F) and we could find no evidence for effects of the transcription factors on proliferation rate. We confirmed that the transduced cells were differentiating to AT1 and AT2 fate in our culture conditions (Fig. 4G,H). As an approximation to the relative production of AT2 versus AT1 cells in these experiments we scored the number of alveolar GFP^+^ cells that co-expressed high levels of pro-SFTPC (Fig. 4I,J). There was no evidence for a change in the percentage of GFP^+^ pro-SFTPC^+^ co-expressing cells when CITED2 or STATs were overexpressed. However, as a positive control for this assay, NOTCH1^ICD^ reduced AT2 cell differentiation, consistent with published data (Guseh et al., 2009).

The effects of STAT proteins on alveolar location of GFP^+^ cells in these experiments could either be mediated through an undetected change in the differential proliferation rate between transduced bronchiolar versus alveolar progenitors, or through promotion of alveolar fate. Analysis of STAT6 protein location in wild-type embryonic lungs showed that it was predominantly bronchiolar. We therefore made lung epithelial-specific deletions of *Stat3* and *Stat5* to test if they were required for alveolar development.

### Lung epithelial-specific knockout of *Stat3* results in a short delay in alveolar development.

**Fig. 5.**
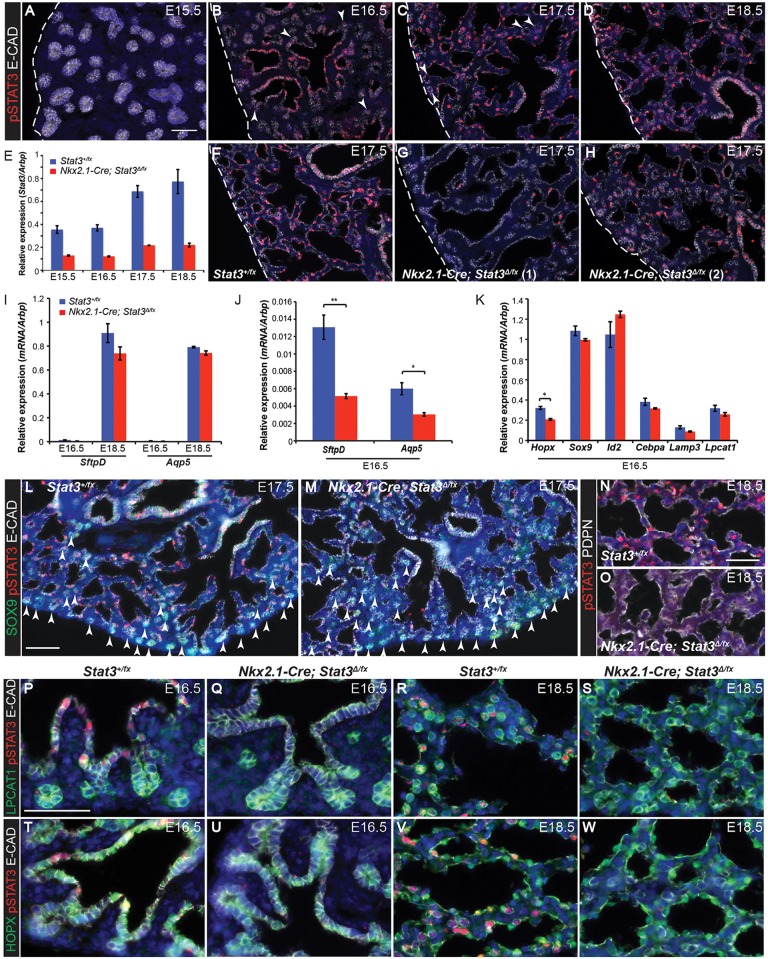
**Lung epithelial specific knock-out of *Stat3* results in a brief delay in lung development.** (A-D) Sections of E15.5, 16.5, 17.5 and 18.5 wild-type mouse lungs stained to show phosphorylated (active) STAT3 protein. Red, pSTAT3; white, E-CAD (epithelium). Arrowheads, distal tips. (E) RT-qPCR for *Stat3* in *Nkx2.1-Cre; Stat3^Δ/fx^* and sibling *Stat3^fx/+^* lungs. Five lungs of each genotype were collected from three independent litters. (F-H) pSTAT3 staining in sibling control (F) and *Stat3* cKO (G,H) lungs shows the highly variable extent of recombination. Red, pSTAT3; white, E-CAD (epithelium). (I-K) RT-qPCR from *Nkx2.1-Cre; Stat3^Δ/fx^* and sibling *Stat3^fx/+^* lungs. Mature differentiation markers *SftpD* and *Aqp5* at E16.5 and E18.5 (I,J) and late progenitor and/or early differentiation markers at E16.5 (K). (L-W) Sections of *Stat3* cKO and sibling lungs. (L,M) E17.5 sections stained for: green, SOX9 (tip progenitors); red, pSTAT3; white, E-CAD (epithelium). Arrowheads, distal tips. (N,O) E18.5 sections stained for: red, pSTAT3; white, PDPN (type 1 cells). (P-S) Green, LPCAT1 (late tip progenitors and type 2 cells); red, pSTAT3; white, E-CAD (epithelium) at E16.5 (P,Q) and E18.5 (R,S). (T-W) Green, HOPX (type 1 cells); red, pSTAT3; white, E-CAD (epithelium) at E16.5 (T,U) and E18.5 (V,W). Blue, DAPI. Dashed line, edge of lung. Scale bars: 50 μm, except 100 μm in L,M. Error bars represent s.e.m. *P<0.05; **P<0.01 by two-tailed *t*-tests with unequal variance; *n*=5.

A detailed time-course of phosphorylated-STAT3 (active, pSTAT3) expression showed that it was undetectable at E15.5, but appeared weakly in the tip progenitors from E16.5 (arrowheads in Fig. 5B,C) and more strongly in differentiating alveolar cells that had exited the distal progenitor pool (Fig. 5A-D; Fig. S4). pSTAT3 was also observed in the bronchioles, consistent with a role in airway differentiation (Tadokoro et al., 2014). The majority of pSTAT3^+^ cells were also positive for E-cadherin (E-CAD).

We generated *Nkx2.1-Cre; Stat3^Δ/fx^* conditional knockout (hereafter *Stat3* cKO) and control *Nkx2.1-Cre; Stat3^+/fx^* (hereafter *Stat3^+/fx^*) embryos to remove STAT3 specifically from the developing lung epithelium. qRT-PCR showed that *Stat3* mRNA levels were decreased in the *Stat3* cKO lungs compared with controls, but recombination was not completely efficient (Fig. 5E). pSTAT3 immunostaining in E17.5 *Stat3* cKO and control lungs showed that recombination in the *Stat3* cKO lungs was highly variable, with some having an almost complete loss of pSTAT3 and others being largely unaffected (Fig. 5F-H). Examination of reporter gene expression in *Nkx2.1-Cre; Rosa26R-fGFP* lungs gave a similar, highly variable, result (Fig. S4A).

To assess a wide range of alveolar fate and differentiation markers we performed qRT-PCR from *Stat3* cKO and control lungs at E16.5 and E18.5. At E16.5, lungs from *Stat3* cKO mice exhibited greater than twofold decreases in the levels of *SftpD* and *Aqp5*, previously reported as markers of mature AT1 and AT2 cells, respectively (Desai et al., 2014) (Fig. 5I,J). However, *SftpD* and *Aqp5* levels were normal at E18.5 and other markers did not change significantly (Fig. 5I-K; Fig. S4C). These results are suggestive of a short delay in alveolar differentiation. To confirm this, we performed a detailed antibody staining time-course in *Stat3* cKO and control lungs, co-staining with pSTAT3 to assess the extent of recombination. We observed a modest increase in the number of SOX9^+^ distal tips in the *Stat3* cKO lungs at E17.5 [Fig. 5L,M; 0.1±0.01 versus 0.18±0.03 tips per 200 μm^2^ (means±s.e.m.), *n*=3 lungs], but tip number seemed normal by E18.5. Expression of the key alveolar fate and differentiation markers commenced at the expected times, with upregulation in differentiating cells by E18.5 (Fig. 5N-W; Fig. S4D-H). These subtle phenotypes are consistent with a brief developmental delay in the *Stat3* cKO lungs.

STAT5A is expressed strongly in a subset of distal tip progenitor cells at E16.5 (Fig. S5A). We generated *Nkx2.1-Cre; Stat5^Δ/fx^* (hereafter *Stat5* cKO) and control *Nkx2.1-Cre; Stat5^+/fx^* (hereafter *Stat5^+/fx^*) embryos to remove STAT5A and STAT5B from the developing lung epithelium. Similar to *Stat3*, qRT-PCR confirmed the partial deletion of *Stat5* (Fig. S5B). However, we were unable to detect any phenotype in the *Stat5* cKO lungs (Fig. S5B-F). This indicates that STAT5 is not required for alveolar fate specification or differentiation. These experiments also provide useful controls showing that the subtle phenotypes observed in the *Stat3* cKO lungs were specific to *Stat3* deletion and not a result of non-specific Cre activity.

### Widespread STAT3 activation accelerates lung alveolar differentiation

**Fig. 6.**
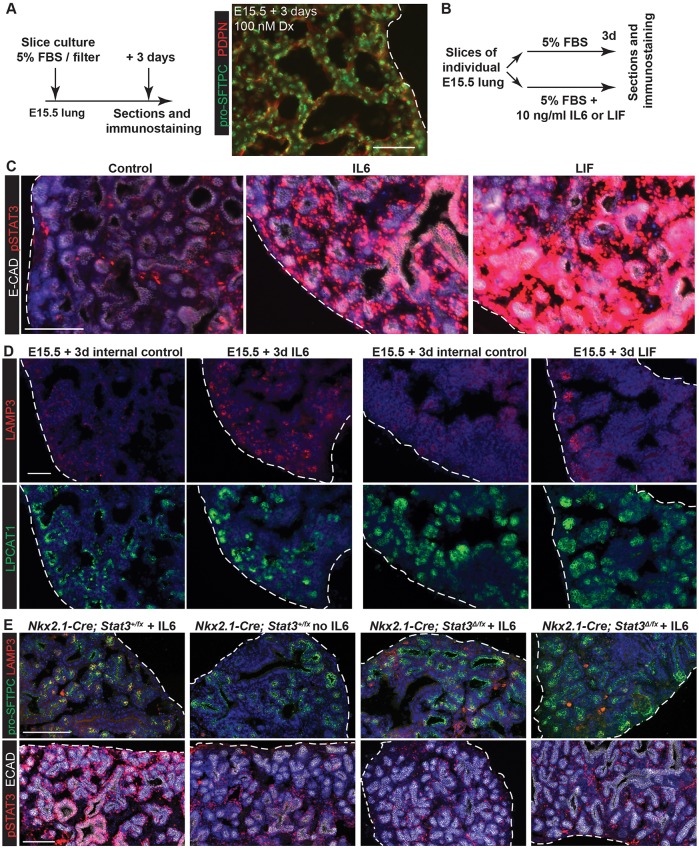
**Ectopic IL6 family ligands result in accelerated AT2 differentiation via STAT3 activation.** (A) Schematic and section of E15.5 slice culture resulting in differentiation of mature saccules with AT1 and AT2 cells in the presence of Dx. Green, pro-SFTPC; red, PDPN. (B) Schematic of IL6 and LIF experiments. Slices from individual lungs were split between two conditions for internal controls. (C,D) Sections from control, IL6- and LIF-exposed wild-type lungs. (C) Red, pSTAT3; white, E-CAD (epithelium). (D) Red, LAMP3 (differentiating AT2 cells); green LPCAT1 (late tip and AT2 cells). (E) Sections from control (*Nkx2.1-Cre; Stat3^+/fx^*) and mutant (*Nkx2.1-Cre; Stat3^Δ/fx^*) lungs with and without IL6. *n*=9 *Nkx2.1-Cre; Stat3^Δ/fx^* lungs analysed in three independent experiments. Top panels: green, pro-SFTPC; red, LAMP3. Lower panels: red, pSTAT3; white, E-CAD (epithelium). Blue, DAPI. Dashed line, edge of lung. Scale bars: 50 μm in A,D; 100 μm in C,E.

We asked if STAT3 activation by ectopic ligand was sufficient to promote alveolar differentiation. We established a culture system in which E15.5 wild-type lung slices were incubated at the air-liquid interface in medium containing 5% FBS. In the presence of Dx robust alveolar differentiation was observed after 3 days, with distinct AT1 and AT2 cells arranged around saccular structures (Fig. 6A). To test the ability of the interleukin 6 (IL6) family ligands, IL6 and LIF, to induce alveolar differentiation, individual lung slices were split and incubated with or without the cytokine for 3 days (Fig. 6B). These experiments were performed with and without Dx. Both IL6 and LIF treatment robustly activated STAT3 throughout the lung slices (Fig. 6C). No detectable phenotypic changes were induced by IL6 and LIF in the presence of Dx. In the absence of Dx, control lung slices showed low levels of LPCAT1 and LAMP3, indicating that alveolar fate specification had occurred. However, there was no evidence of AT2 differentiation or saccule formation. When either IL6 or LIF was added in the absence of Dx, levels of the more specific AT2 differentiation marker LAMP3 were much higher compared with controls (Fig. 6D; 9/9 IL6-treated and 6/7 LIF-treated lungs in three independent experiments). HOPX and SOX9 expression were unchanged (Fig. S6). This suggests that AT2 differentiation occurred in response to cytokine signalling, but the overall morphology of the slices remained immature. The effect of IL6 upon AT2 differentiation was consistently more potent than LIF. Oncostatin M (*Osm*) and its receptor (*Osmr*) were also detected in our microarray experiments (Fig. 4B), but addition of recombinant OSM to the slice cultures did not promote LAMP3 expression (Fig. S6; 0/6 OSM-treated lungs in two independent experiments).

We tested if STAT3 is required to mediate the effects of IL6 in the slice cultures. We cultured *Stat3* cKO and sibling control lungs with and without IL6 and observed that in the absence of STAT3, LAMP3 levels were reduced to those of control lung slices with no IL6 exposure (Fig. 6E). These results suggest that activation of STAT3 signalling by IL6 or LIF is sufficient to promote expression of mature AT2 markers, even in the absence of glucocorticoids.

### GR and STAT3 signalling act in parallel during alveolar development

We considered the possibility that STAT3 and GR signalling act redundantly in alveolar development and that inactivation of both signalling pathways would result in a stronger phenotype. To test this hypothesis we cultured E12.5 *Stat3* cKO and littermate control lungs for 5 days in the absence of Dx, or in the presence of a glucocorticoid and progesterone antagonist mifepristone, so that both STAT3 and GR signalling would be decreased (Fig. S7). In both conditions, alveolar fate markers were expressed as expected (Fig. S7) and whole-mount analysis indicated that the transition from producing bronchiolar- (SOX2^+^) to alveolar-fated (SOX2^−^) descendants occurred at the same time in all genotypes (Fig. S7D). These results suggest that STAT3 and GR signalling are not acting redundantly at these stages of alveolar development.

**Fig. 7.**
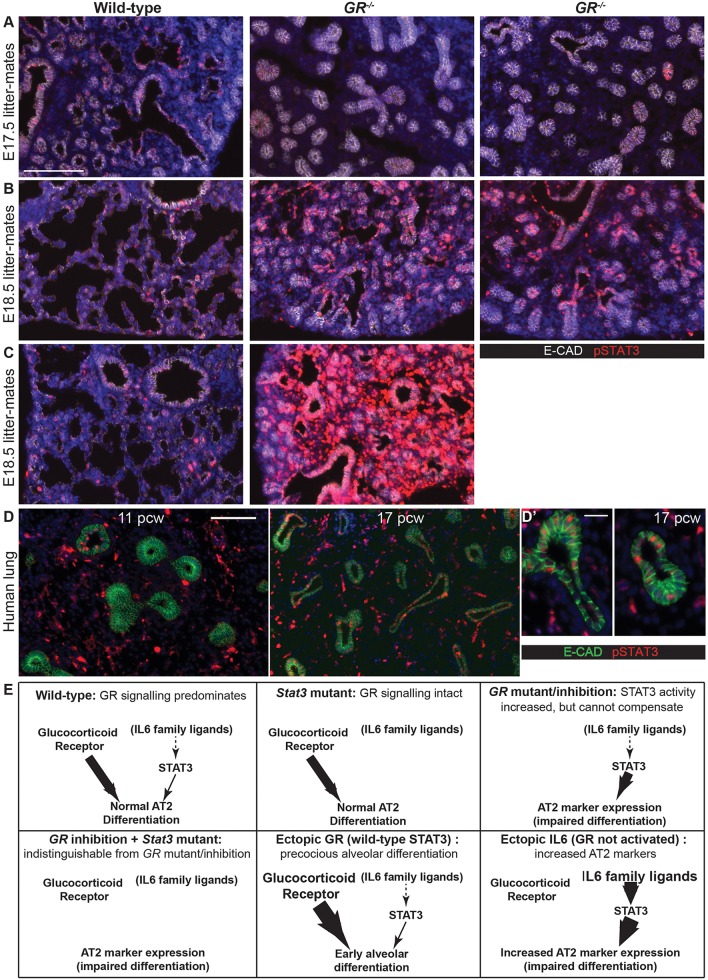
**STAT3 and glucocorticoid function co-operatively in alveolar differentiation.** (A-C) Cryosections of *GR^−/−^* and littermate lungs at E17.5 (A), E18.5 (B) and in an independent litter at E18.5 (C). Red, pSTAT3; white, E-CAD. A total of five *GR^−/−^* and five *GR^+/+^* sibling lungs from three independent litters were observed at both E17.5 and E18.5. (D) Human embryonic lung sections from 11 and 17 pcw. Green, E-CAD (epithelium); red, pSTAT3; blue, DAPI. Note the presence of mesenchymal background staining in the 11 and 17 pcw human samples. (E) We propose that in wild-type lung development STAT3 and GR signalling work in parallel to promote alveolar differentiation. GR has the predominant role indicated by subtle phenotypes in the *Stat3* mutant and impaired differentiation, but only slightly delayed alveolar specification in the *GR* mutant. Loss of both pathways does not result in a greater phenotype, indicating that the ectopic pSTAT3 observed in the *GR* mutants is not sufficient to compensate. Nevertheless, ectopic activation of either pathway is sufficient to promote aspects of alveolar differentiation with the exact effects depending on timing. However, given that neither pathway is absolutely necessary for the initiation of alveolar differentiation to occur, other signalling mechanisms must also be involved. Scale bars: 100 μm A-D, 20 μm D′.

Our ectopic ligand results (Fig. 6) are consistent with STAT3 acting downstream of GR signalling to mediate some of its effects. We therefore examined pSTAT3 expression in *GR^−/−^* and littermate control lungs. At E17.5 the *GR^−/−^* lungs had very little pSTAT3 expression, consistent with their developmental delay (Fig. 7A). However, by E18.5 the pSTAT3 signal was reproducibly greater in the *GR^−/−^* lungs compared with the controls (Fig. 7B). This effect was even more pronounced in an independent litter, which was collected at a slightly later developmental stage (Fig. 7C).

The increase in pSTAT3 levels could also be replicated by treating cultured lungs with the glucocorticoid and progesterone antagonist mifepristone (Fig. S8A). We considered the hypothesis that the increase in pSTAT3 in the *GR* mutants was related to an increase in apoptotic cell death, but were unable to detect a significant increase in the levels of apoptosis measured by cleaved caspase 3 staining (Fig. S8B). Interestingly, we observed an increase in LIF protein levels in the *GR^−/−^* lungs, possibly partly explaining the increase in pSTAT3 (Fig. S8C).

These data suggest that STAT3 signalling is not directly downstream of GR signalling in a linear pathway. Rather, it is likely that STAT3 and GR signalling act in parallel to promote the differentiation of alveolar cells, but that there is some cross-regulation between the two pathways. Like GR, STAT3 is not absolutely required for alveolar differentiation (Fig. 3). However, ectopic exposure to STAT3 activating ligands was sufficient to promote AT2 differentiation (Fig. 6). Activation of STAT3 signalling could be useful for promoting the maturation of human induced pluripotent stem cell (iPSC)-derived alveolar epithelium, or for human lung regeneration. We therefore examined human embryonic lungs to test whether STAT3 and GR signalling are active at similar stages to mouse lung development (Fig. 7D). We were unable to detect epithelial pSTAT3 in the distal epithelium of pseudoglandular stage human lungs [post conception weeks (pcw) 11 and 14 were tested]. However, pSTAT3 was detected strongly in the epithelium of a 17 pcw lung, which had a much more canalicular appearance. We therefore hypothesize that STAT3 signalling plays similar roles in mouse and human lung alveolar development, but further investigation is required to test this idea. By contrast nuclear (active) GR was slightly different between the two species. It was detected strongly in both mesenchyme and epithelium of E16.5 and E18.5 mouse lung as previously reported (Fig. S9A-C), though not at E14.5. However, nuclear GR was already present in the mesenchyme of 8 pcw human embryonic lungs and the distal tip epithelium of 11 and 17 pcw samples (Fig. S9D-F).

## DISCUSSION

Elucidating the cellular and molecular mechanisms that control the induction of alveolar fate in the distal tip progenitors of the embryonic lung, and subsequent alveolar differentiation, will be important for efforts to regenerate the alveolar epithelium. Our grafting experiments have conclusively shown that extrinsic signalling from the surrounding tissue is sufficient to control the fate of the progeny produced by distal lung epithelial progenitors. This is analogous to previous experiments that showed that mesenchymally derived signals were sufficient to impose a tracheal, or lung, branching pattern on embryonic lung endoderm (Alescio and Cassini, 1962; Shannon, 1994; Shannon et al., 1998). Interestingly, the differentiating stalk cells were surprisingly plastic and could be induced to produce alveolar-fated descendants, even in the absence of exogenous Dx. The E12.5 stalks were more plastic than their more differentiated E16.5 counterparts. The mechanisms underlying this plasticity, particularly the extent of any epigenetic changes, will be an interesting topic of future study.

We present evidence that STAT3 and GR signalling are individually sufficient to promote alveolar differentiation and that they act in parallel during normal embryonic lung development (Fig. 7E). Our results also suggest that lung alveolar initiation is a highly robust process during which the GR and STAT3 pathways are redundant with other, yet to be identified, signalling modules. We observed that STAT3 and STAT5A have similar expression patterns and modest lung phenotypes, with one possibility for this being that they are redundant in alveolar development, although we could find no evidence for compensatory upregulation or activity of either protein (Fig. S4G; Fig. S5F). The two genes are located adjacently on chromosome 11 and testing this hypothesis will require the development of additional tools for gene deletion.

We focus on the initial stages of lung alveolar differentiation and show that neither STAT3 nor GR are absolutely required, individually or redundantly together, for this process. Nevertheless, experimental activation of either pathway can promote distal progenitor alveolar fate, or AT2 differentiation, depending on timing. Glucocorticoid levels rise dramatically in the mouse foetus following onset of steroidogenesis at ∼E15. We propose that during normal mouse lung development both STAT3 and GR signalling promote alveolar differentiation from ∼E16.5 onwards (Fig. 7E). GR has a greater role and is also absolutely required for the later stages of alveolar differentiation (Cole et al., 2004). However, both pathways are redundant with other signalling mechanisms at the early stages of alveolar differentiation. When GR signalling is disrupted we observe that signalling via STAT3 is increased, probably in an attempt to compensate. In support of this idea, ectopic STAT3 activity *in vitro* can promote AT2 differentiation in the absence of exogenous Dx. Interestingly, GR and STAT proteins have been reported to act together in multiple settings via several molecular mechanisms including joint transcriptional activation and/or repression (Engblom et al., 2007; Langlais et al., 2012) and control of nuclear localisation (Shin and Reich, 2013).

Finally, we present evidence that STAT3 and GR signalling are active at the canalicular stage of normal human embryonic development, supporting the idea that their functions are conserved across species. It will be important to test if manipulating STAT3 can promote improved AT2 differentiation in human lung iPSC-derived cultures, or even directly in the lungs of premature infants. Our data show that alveolar fate determination is a highly robust process, probably involving additional extrinsic signalling inputs as well as STAT3 and GR. It will be important to define these pathways for human and mouse embryonic lungs.

## MATERIALS AND METHODS

### Animals

All experiments were approved by University of Cambridge and University of Edinburgh local ethical review committees and conducted according to Home Office project licences PPL80/2326, 70/812 and 70/7874. Mouse strains *Rosa26R-mT/mG* (Muzumdar et al., 2007), *Nr3c1^Gt(ESKN92)Hgs^* (*GR* null) (Michailidou et al., 2008), *Stat3^fx^* (Alonzi et al., 2001), *Stat5^fx^* (Cui et al., 2004), *Nkx2.1-Cre* (Xu et al., 2008b), *Id2-CreER* (Rawlins et al., 2009a) and *Rosa26R-fGFP* (Rawlins et al., 2009b) have been described. *Stat3^Δ/+^* and *Stat5^Δ/+^* were generated by crossing floxed alleles to *Zp3-Cre* (de Vries et al., 2000). Transgenic strains were maintained on a C57Bl/6J background (at least N4 back-crosses, or >20 back-cross generations for *GR^+/−^*). Wild-type mice were outbred MF1 strain.

### Human material

The human embryonic and foetal material was provided by the Joint MRC/Wellcome Trust (grant 099175/Z/12/Z) Human Developmental Biology Resource (www.hdbr.org), or collected at Addenbrooke's Hospital (Cambridge, UK) under permission from NHS Research Ethical Committee (96/085). Samples used had no known genetic abnormalities.

### Lung cultures and manipulations

All *in vitro* cultures were performed in at least three independent experiments unless otherwise stated. Mouse E12.5 lungs were cultured on Whatman Nucleopore filters 10 μm pore size (Millipore) in BGJ/b medium (Sigma) at 37°C, 5% CO_2_ up to 8 days. E15.5 lungs were cut into slices using a razor blade and cultured up to 3 days on filters in BGJ/b medium with 5% FBS (Gibco). Dexamethasone (Dx; Sigma) was used at 50 nM for E12.5 lungs and 100 nM for E15.5 slices. Mifepristone (Sigma) was used at 3 μM, recombinant IL6 (R&D Systems) and LIF (Millipore) at 10 ng/ml and recombinant OSM (R&D Systems) at 25 ng/ml.

For grafting, tip and stalk cells were microdissected from *Rosa26R-mT/mG* heterozygous embryos using tungsten needles following 5 min in Dispase (Gibco, 16 U/ml final concentration) at room temperature. Stalk cells were taken from a region of future bronchiole 2-3 branches above the distal tip. Microdissected epithelial tips and stalks were washed in phytohemagglutin (PHA-P, lectin from *Phaseolus vulgaris*; Sigma, 0.2 mg/ml final concentration) and inserted into a pocket made in the mesenchyme of a wild-type E12.5 lung on a filter. Grafts were always placed in approximately the centre of the host lung.

For adenovirus injections, E12.5 lungs were submerged in PBS and microinjected with 2×10^10^ infectious units (IFU)/ml virus mixed with Trypan Blue (4:1 ratio). The lumen of the branching tree was filled (∼10 nl) by microinjecting with a Nanoject II Auto-Nanoliter Injector. Lungs were incubated at room temperature in PBS at least 1 h before transfer to filter for culturing.

### Immunostaining

E15.5-18.5 mouse lungs were fixed 1-2 h in 4% paraformaldehyde at 4°C. Human lungs were fixed overnight. Samples were washed in PBS, sucrose protected, embedded in optimum cutting temperature compound (OCT; Tissue Tek) and sectioned at 8 μm. Primary antibodies: acetylated tubulin (mouse, 1:3000, Sigma, T7451), CEBPA (rabbit, 1:500, Santa Cruz, sc-61), cleaved caspase 3 (rabbit, 1:100, Abcam, ab2302), E-CAD (rat, 1:1000, Invitrogen, 13-1900; or mouse, 1:1000, BD Biosciences, 610182), GFP (chick, 1:1000, Abcam, AB13970), GR (rabbit, 1:100, Santa Cruz, sc-1004), HOPX (rabbit, 1:50, Santa Cruz, sc-30216, clone FL-73), KI67 (mouse, 1:200, BD, 550609), LAMP3 (rat, 1:100, Dendritics, DDX0192, clone 1006F7.05), LIF (goat, 1:100, R&D Systems, AB-449-NA), LPCAT1 (rabbit, 1:500, Proteintech), PDPN (hamster, 1:1000, DSHB, 8.1.1), RFP (rabbit, 1:250, Rockland, 600-401-379), pro-SFTPC (rabbit, 1:500, Millipore, AB3786), SOX2 (goat, 1:250, Santa Cruz, sc-17320, clone Y-17), SOX9 (goat, 1:200, R&D Systems, AF3075), pSTAT3-Tyr705 (rabbit, 1:200, Cell Signaling, 9145), STAT5a (rabbit, 1:20, Abcam, ab7968). Antigen retrieval was by boiling in 10 mM sodium citrate, pH 6 for mouse anti-E-CAD, rabbit-anti-HOPX, mouse-anti-KI67, goat-anti-SOX2, rabbit-anti-pSTAT3. Alexa Fluor-conjugated secondary antibodies (1:2000, Life Technologies; see Table S2 for details). DNA (DAPI, Sigma). Mounting in Fluoromount (Sigma).

pSTAT3 was amplified using the TSA Plus Cyanine 3 Kit (PerkinElmer, NEL744001KT). For double rabbit primary staining (HOPX and LPCAT1; HOPX and pSTAT3; HOPX and SOX2; LPCAT1 and pSTAT3; CEBPA and pro-SFTPC) an excess (40 μg/ml) of Fab fragment donkey anti-rabbit IgG (H+L) (Jackson ImmunoResearch, 711-007-003) was used to block the first primary antibody.

For whole mounts, cultured E12.5+5 days mouse lungs were fixed 2 h in 4% paraformaldehyde at 4°C, washed in PBS with 2% non-fat milk powder and 0.2% Triton X-100 and stained for E-CAD (rat, 1:1000, Invitrogen, 13-1900), SOX2 (goat, 1:250, Santa Cruz, sc-17320, clone Y-17) or SOX9 (rabbit, 1:1000, Millipore, AB5532). Samples were passed through a glycerol series before mounting in Vectashield (Vector Labs).

### Microscopy and image scoring

Slides were imaged on a Zeiss AxioImager compound microscope, or Olympus FV1000 confocal microscope where stated. Cell numbers were scored manually in Fiji (ImageJ, NIH). Protein expression levels in Fig. 1 were quantified using a custom macro for ImageJ (Schneider et al., 2012) (see supplementary Materials and Methods) to measure the area of signal in each channel and calculate the proportion of the cell area containing signal for each. The DAPI channel was Gaussian blurred and Huang thresholded to give a representation of the cell area from the nuclear signal, other channels were maximum entropy thresholded (Kapur et al., 1985) to give the area containing signal above background. This gave a reliable metric for assessing the relative number of cells positive for each marker, which matched our visual assessment.

For grafting experiments, serial sections were cut through the entire lung and even-numbered slides stained to show bronchiolar fate and odd-numbered for alveolar fate. Grafts were tracked in each section and scored as bronchiolar, alveolar, or mixed. Grafts were scored as mixed if they contained two clearly separated bronchiolar and alveolar regions each greater than 10 cells. Statistical tests were two-tailed Fisher's exact tests.

For adenovirus experiments, epithelial cells were scored based on their location and E-CAD staining as bronchiolar (columnar, lower intensity) or alveolar (squamous, higher intensity) and for the presence or absence of nuclear GFP. The normalised GFP^+^ alveolar:bronchiolar ratio was calculated by

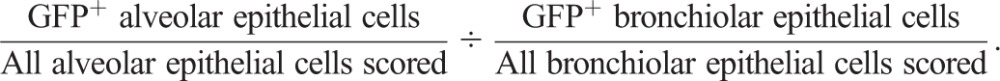

GFP^+^ alveolar cells were later scored for the presence or absence of pro-SFTPC in images taken with a defined exposure time. Images were scored by two independent investigators who were blind to the experimental group. Statistical tests were two-tailed *t*-tests with unequal variance.

### RT-qPCR and microarrays

Total RNA was extracted using Qiagen RNeasy Mini Kit and cDNA was synthesised using Superscript III reverse transcriptase (Life Technologies). Primer sequences: *Aqp5*, 5′-AGGTGTGTTCAGTTGCCTTCTTC-3′ and 5′-AGATGAGGGTGGCCAGGAA-3′; *Cebpa*, 5′-GAGCTGAGTGAGG-CTCTCATTCT-3′ and 5′-TGGGAGGCAGACGAAAAAAC-3′; *Hopx*, 5′-TGCCTTCGGAATGCAGATCT-3′ and 5′-AGCTCAAGGGCCTGGCTC-3′; *Id2*, 5′-AAGGTGACCAAGATGGAAATCCT-3′ and 5′-CGATCTGCAGGTCCAAGATGT-3′; *Lamp3*, 5′-AATGTGAACGAGTGTTTGTCTGACTA-3′ and 5′-GACGACCACGATGATTGCAA-3′; *Lpcat1*, 5′-TTATGGAGGAAGGTCGTGGACTT-3′ and 5′-GAAGCCGCCAGCAAACC-3′; *SftpD*, 5′-AGCAGAAATGAAGAGCCTCTCG-3′ and 5′-AGGGTGCAGGTGTTGGGTAC-3′; *Sox9*, 5′-GCAGCACTGGGAACAACCA-3′ and 5′-GCTCTGTCACCATAGCTTTTCTCTT-3′; *Stat3*, 5′-CAGAGGGTCTCGGAAATTTAACAT-3′ and 5′-CTCCCTAAGGGTCAGGTGCTT-3′; *Stat5a*, 5′-TGGCTTTGCACGTTTCACA-3′ and 5′-CACCGCTTTAGCCACAAACC-3′.

For microarray analysis E11.5 tip cells were manually microdissected. GFP^+^ E17.5 tip cells, with a small number of their immediate progeny, were collected by flow cytometry from *Id2-CreER; Rosa26R-fGFP* embryos exposed to tamoxifen at 150 μg/gram mother's body weight at E16.5. RNA was isolated using the Qiagen RNeasy Mini Kit, amplified and labelled using the Ovation RNA Amplification Kit V2 and FL-Ovation Biotin V2 (NuGEN). Hybridisation to Affymetrix mouse 430.2 microarray chips (five chips per condition). Data were analysed using the Bioconductor Package in R (https://www.r-project.org/; R Team, 2014). Gene Ontology analysis using GOToolbox (http://genome.crg.es/GOToolBox/). Raw data was deposited in GEO (http://www.ncbi.nlm.nih.gov/geo) under accession number GSE75860.

### Adenovirus production

Adenovirus construction was as previously described (Zhou et al., 2008). Genes were cloned from E16.5 lung cDNA into a shuttle vector containing an internal ribosome entry site linked to nuclear GFP (*IRES-nGFP*), and then into the pAd/CMV/V5-DEST adenoviral vector (Invitrogen). High titre non-replicating virus (>1×10^10^IFU/ml) was obtained by purification with the Fast-Trap Adenovirus Purification and Concentration Kit (Millipore, FTAV00003). Titre was determined using AdEasy Viral Titer Kit (Agilent Technologies, 972500). Two isoforms of *Stat3* and *Stat5a* were cloned from E16.5 wild-type lungs, both were used (*Stat3.1/Stat3.2*; *Stat5a.1/Stat5a.2*) with indistinguishable results.
